# Disaster in pregnancy: midwifery continuity positively impacts infant neurodevelopment, QF2011 study

**DOI:** 10.1186/s12884-018-1944-5

**Published:** 2018-07-27

**Authors:** Gabrielle Simcock, Sue Kildea, Sue Kruske, David P. Laplante, Guillaume Elgbeili, Suzanne King

**Affiliations:** 1 Mater Research Institute-University of Queensland, Brisbane, QLD Australia; 2 School of Psychology, The University of Queensland, Brisbane, QLD Australia; 3 School of Nursing, Midwifery, and Social Work, The University of Queensland, Brisbane, QLD Australia; 4 Institute of Urban Indigenous Health, Brisbane, QLD Australia; 5 Schizophrenia and Neurodevelopmental Disorders Research, Douglas Mental Health Institute, 6875 LaSalle Boulevard, Verdun, Quebec, H4H 1R3 Canada; 6 Department of Psychiatry, McGill University, Montreal, QC Canada

**Keywords:** Midwifery group practice, Prenatal maternal stress, Infant development

## Abstract

**Background:**

Research shows that continuity of midwifery carer in pregnancy improves maternal and neonatal outcomes. This study examines whether midwifery group practice (MGP) care during pregnancy affects infant neurodevelopment at 6-months of age compared to women receiving standard hospital maternity care (SC) in the context of a natural disaster.

**Methods:**

This prospective cohort study included 115 women who were affected by a sudden-onset flood during pregnancy. They received one of two models of maternity care: MGP or SC. The women’s flood-related objective stress, subjective reactions, and cognitive appraisal of the disaster were assessed at recruitment into the study. At 6-months postpartum they completed the Ages and Stages Questionnaire (ASQ-3) on their infants’ communication, fine and gross motor, problem solving, and personal-social skills.

**Results:**

Greater maternal objective and subjective stress predicted worse infant outcomes. Even when controlling for maternal stress from the flood, infants of mothers who were in the MGP model of maternity care performed better than infants of mothers in SC on two of the five ASQ-3 domains (fine motor and problem solving) at 6-months of age. Furthermore, infants in the SC model were more likely to be identified as at risk for delayed development on these domains than infants in the MGP model of care.

**Conclusions:**

Continuity of midwifery care has positive effects on infant neurodevelopment when mothers experience disaster-related stress in pregnancy, with significantly better outcomes on two developmental domains at 6 months compared to infants whose mothers received standard hospital care.

## Background

Women experiencing continuity of midwifery carer during the maternity episode, in comparison to those in standard care, have better maternal, childbirth, and neonatal outcomes including reduced interventions (e.g., induction, analgesia, episiotomy) and operative birth (e.g., instrumental, caesarean) [1]. There are also significant reductions in preterm births [2, 3], fetal loss (before and after 24-weeks gestation) and neonatal death [3], and admissions to special care nurseries, including for infants born to mothers with risk factors [1, 2]. To our knowledge, however, whether the benefits of midwifery continuity also extend to infant developmental outcomes has not been explored; although nurse home-visits in pregnancy, which continue up to 2-years postpartum, can benefit infant development [4].

Prior research shows that stress in pregnancy has an enduring influence on neonatal outcomes [5, 6] and infant neurodevelopment, including adversely affecting early cognitive [7], linguistic [8], motor [9], and behavioral [10] development. The current study is part of the prospective longitudinal Queensland Flood study (QF2011) examining the effects of stress in pregnancy on infant development [11]. Women in the QF2011 study were recruited from a tertiary hospital where approximately half the women were receiving standard hospital care (SC) and half were receiving midwifery group practice (MGP) maternity care. SC is provided by rostered and on-call doctors and midwives that may be shared with a general practitioner in the community. In MGP care, women have a primary midwife, with two to three back-up midwives working in a small group.

Women in the QF2011 study were pregnant when a sudden onset flood severely affected large portions of the state of Queensland, Australia in January 2011. At 6-months postpartum the flood-affected women rated their infants’ neurodevelopment using the Ages and Stages Questionnaire-3 (ASQ-3 [12]) [9, 13]. With high levels of flood-related prenatal maternal stress (PNMS) girls had poorer problem solving skills than boys, and flood-exposure in late pregnancy predicted worse personal-social skills; yet there were no effects of PNMS on infant communication [13]. Furthermore, higher levels of PNMS or a negative appraisal of the flood, predicted poorer gross and fine motor development; and infants had worse motor scores when the flood occurred in late-pregnancy [9].

As flood-related PNMS has negative effects on infant neurodevelopment at 6-months, we examined whether the model of maternity care (MGP vs SC) that the women received would buffer their infants’ development from the effects of flood-related PNMS. We hypothesized that infants born to mothers who received MGP care would have higher scores on the ASQ-3 scales compared to SC infants when controlling for maternal flood-exposure in pregnancy, and would be less likely to be identified as at risk for developmental delay than SC infants.

## Methods

### Sample composition

**Fig. 1:**
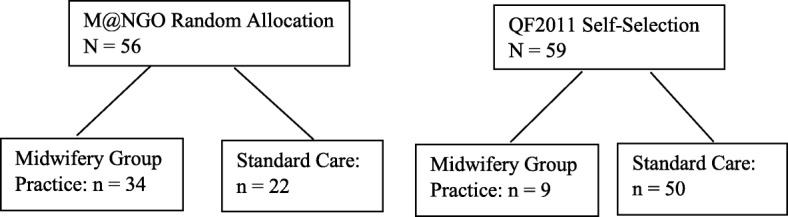
The number of women in the final sample from the randomized control trial Midwifery @ New Group Options (M@NGO) and the 2011 Queensland Flood Study (QF2011) who were in the midwifery group practice vs standard care models of maternity care

QF2011 included two groups of women who were pregnant during the flood and recruited from the flood affected region. The first group was comprised of pregnant women enrolled in a randomized control trial (RCT; M@NGO study) assessing the effectiveness of MGP over SC [14], if eligible to join QF2011 [11]. A second group were recruited specifically into QF2011. QF2011 recruitment eligibility included women over 18-years of age who were fluent in English with singleton pregnancies at the time of the flood. M@NGO women were randomly allocated to MGP or SC whereas QF2011 women self-selected their model of care or received SC when all MGP places were filled. 56 women were recruited from M@NGO and 59 women were recruited into QF2011. In the final combined sample (*N* = 115), there were 43 women in MGP and 72 in SC. The flow-chart in Fig. 1 shows the number of women in MGP and SC for each care-type. See the published protocols [11, 14] for full recruitment details.

Maternal demographics and flood-related prenatal stress were assessed via self-report questionnaires at recruitment into the study (April 2011 to January 2012). Maternal mental health and infant development were assessed via questionnaires at 6-months postpartum. Data from eight infants were excluded from analyses as the questionnaires were completed outside of the accepted age-range (6-months +/− 1-month) and data from three infants were excluded due to preterm birth (< 36-weeks gestation). The final sample included data from 115 6-month-olds (see Table 1). The study received ethical approval from the Hospital Review Board (M@NGO: 0805072 M; QF2011: 1709 M) and University Review Board (#2013001236).

**Table 1:** Sample compositions in Midwifery (MGP) and Standard (SC) care groups

Variable	MGP (*N* = 43)	SC (*N* = 72)	
	Mean (*SD*)	Mean (*SD*)	*p* value
Objective flood stress	19.81 (15.34)	21.51(17.72)	0.60
Subjective flood stress	0.06 (1.04)	0.02 (1.06)	0.86
Maternal age at infant birth	30.61 (4.37)	32.46 (4.63)	0.04
Socioeconomic status	1046.47 (51.02)	1054.83 (61.10)	0.45
Years education	14.77 (1.76)	14.62 (1.69)	0.69
Maternal EPDS score	5.87 (4.01)	5.64 (4.18)	0.77
Birth gestational age (wks)	39.65 (1.15)	39.18 (1.17)	0.04
Birth weight (kgs)	3.67 (0.46)	3.48 (0.44)	0.02
Infant age at assessment (mths)	6.29 (0.05)	6.26 (0.04)	0.56
N	%	%	
Appraisal of flood:			0.02
Negative	23.3	45.1	
Neutral+Positive	76.7	54.9	
Infant sex:			0.75
Boys	48.8	45.8	
Girls	51.2	54.2	
Race:			0.91
Caucasian Australian	97.7	98.5	
Other	2.3	1.5	
Marital status:			0.66
Married/DeFacto	92.3	92.3	
Single/Divorced	7.7	7.7	
Parity			0.026
0	65.1	43.3	
1–2	32.6	50.7	
3+	2.3	6	

### Maternity care models

The MGP model offers continuity of midwifery carer throughout the prenatal, intrapartum and postnatal period up to 6-weeks following birth. Midwives typically carry individual caseloads of 36–40 women per annum, and provide 24/7 telephone access. The midwives work in small groups (*n* = 2–4) with each woman being assigned one primary midwife who is then backed up by the others for leave entitlements. Clinical consultations are provided in the home, in community-based clinics, or in hospital outpatient departments, depending on the needs of the woman. All women labor and birth at the hospital. The high degree of relational continuity, and familiarity and comfort of the domiciliary or community setting, affords multiple opportunities for women to get to know the midwives and establish relationships of trust. Women are visited at home postnatally by their primary midwife for up to 6-weeks.

In SC models, women receive the majority of their care from unfamiliar rostered on-call obstetricians and midwives that may include shared care from community-based general practitioners. Although there may sometimes be high relational continuity in SC, there is generally less continuity and hence limited opportunities for women to build relationships with staff during pregnancy. Women are cared for in labor and birth by clinicians they may not have previously met, as each area of antenatal, intrapartum and postnatal services are usually staffed by different midwives. Labor and birth occur at the hospital with limited postnatal follow-up. Women may receive in-home care if they select early discharge, before 48 h for vaginal birth and 72 h for caesarean section, but is this rarely from a midwife they have previously met.

### Prenatal maternal stress

Woman’s flood-related *objective stress* experience was assessed using a specifically tailored questionnaire based on prior flood PNMS research [15]. Items assessed four key dimensions of stress: threat, loss, scope, and change. Scores on each dimension ranged from 0 (no impact) to 50 (extreme impact) and were summed, giving a total Queensland Flood Objective Stress Score (QFOSS) out of 200; higher scores indicating more severe flood exposure.

Woman’s emotional reaction to the Queensland flood was assessed using a *composite subjective stress* score based on three self-report recruitment questionnaires. The 22-item Impact of Event Scale – Revised (IES-R) [16] assessed women’s emotional responses with scores for post-traumatic-like symptoms relative to the flood over the past seven days. Women rated items on a 0 (not true) to 4 (extremely true) Likert scale. The 13-item Peritraumatic Distress Inventory (PDI-Q) [17] and the 10-item Peritraumatic Dissociative Experiences Questionnaire (PDEQ) [18] retrospectively assessed women’s reactions to the floods at the time it occurred; women rated the statements on 5-point rating scales (‘not true’ to ‘extremely true’). The COmposite Score for MOthers’ Subjective Stress (COSMOSS) was calculated using Principal Component Analysis (PCA) on the three subjective stress questionnaire scores from the 230 participants who provided recruitment PNMS data. The PCA-derived algorithm was: COSMOSS = (0.36*IESR) + (0.40*PDI) + (0.39*PDEQ), explaining 76.27% of the overall subjective stress variance. COSMOSS is standardized with a mean of 0 and standard deviation (*SD*) of 1, such that positive and negative scores represent levels of subjective stress that are higher or lower than the mean, respectively.

Women’s *cognitive appraisal* of the impact of the flood was assessed with the question: “If you think about all of the consequences of the 2011 Queensland flood on you and your household, would you say the flood has been…?” Women rated their appraisal on a 5-point scale, from Very Negative (− 2) to Neutral (0) to Very Positive (+ 2). To differentiate women who appraised the flood as stressful the scores were dichotomized into ‘negative’ appraisal (scored 0) versus ‘neutral or positive’ appraisal (scored 1).

### Infant development

Infant development was assessed using the ASQ-3 [12]. This is a parent-completed screening tool encompassing five domains of infant development: communication, problem solving, gross motor, fine motor, and personal-social skills. Mothers rated their infants’ development for each of the 30 items as ‘yes’, ‘sometimes’ or ‘not yet’ according to whether the infant achieved the described behavior at 6-months of age. The ASQ-3 is normed for each scale; scores below one *SD* on a given scale indicate further monitoring is recommended, whilst specialist assessment is recommended for scores below two *SD* from the mean. The ASQ-3 has high test-retest reliability (correlation coefficients range = 0.75–0.82), good internal consistency (Cronbach alphas range: 0.51–0.87), and high validity to practitioner-administered tools [19].

### Maternal and infant covariates

To control for other factors known to influence infant development, covariates included maternal age at the infants birth, education level, socio-economic status (using the SEIFA based on Australian postcodes; *M* = 1000, *SD* = 50), parity, and postnatal depression at 6-months postnatal using the Edinburgh Postnatal Depression Scale (EPDS) [20]. Infant gestation length and birth size were obtained from hospital records (see Table 1).

### Statistical analyses

Pearson’s correlations examined the associations between infant scores on the ASQ-3 scales and the PNMS variables. We conducted one-way analyses of covariance (ANCOVAS) to compare infant ASQ-3 scores by care type (MGP vs SC), controlling for flood-related objective stress. Hierarchical liner regression analyses examined the ability of PNMS, model of care, and covariates to explain variance in infant scores on the ASQ-3. The models for the regression analyses for the five ASQ-3 scales were: First, objective stress was entered into the model, followed by composite subjective stress in step 2, and cognitive appraisal in step 3. In step 4, model of care was entered, followed by sex of the infant and timing of the flood in gestation in steps 5 and 6, respectively. If there were significant correlations between maternal or infant factors and ASQ-3 scores, they were included in the next 2 steps respectively. To test for any buffering effects of model of care, an interaction term between PNMS and model of care was added in a final step. Because of the relatively small sample size, all models were then trimmed of non-significant variables, except for QFOSS, and the analyses were rerun. Pearson’s chi-squared tests assessed the frequency of infants requiring ongoing monitoring due to risk of delayed development (scoring ≤1*SD* below the standardized mean) in each maternity care model on the ASQ-3 scales. All analyses were conducted using SPSS v22.

## Results

### Sample

Comparison of women who returned infant questionnaires and those who did not showed no significant differences in terms of maternal demographics or PNMS. Descriptives for the sample variables are shown for the MGP and SC groups in Table 1. Between-group comparisons showed that the sample characteristics were not significantly different on many key variables including maternal age, mental health (depression) and demographics (SEIFA and years schooling). However, mothers were more likely to appraise the flood as negative in the SC group vs the MGP group, and infants in the MGP model were born to younger mothers than those in the SC model, and they had longer gestations and were heavier than infants in the SC model. Women in MGP were more likely to be parimiparous than women in SC.

### Ages and Stages scores

The ASQ-3 scores for the infants in the MGP and SC groups are shown in Table 2. Infants of mother’s in the MGP group had significantly higher scores than those in the SC group on the fine motor and problem solving scales; and there were no difference on the other three scales.

**Table 2:** The Ages and Stages-3 (ASQ-3) scores in Midwifery (MGP) and Standard (SC) care groups

ASQ-3 Scale	MGP	SC	*p* value
	M (*SD*)	M (*SD*)	
Communication	48.37 (9.24)	47.01 (9.44)	0.48
Gross Motor	45.35 (11.82)	45.24 (11.01)	0.96
Fine Motor	50.58 (9.40)	45.14 (12.30)	0.015
Problem Solving	53.72 (7.16)	48.39 (11.02)	0.006
Personal-Social	48.84 (9.56)	45.49 (11.57)	0.12

### Correlations

Table 3 shows the correlations between PNMS variables and ASQ-3 scores. Gross motor, fine motor, and problem solving were significantly negatively correlated with objective hardship, such that higher objective stress predicted poorer scores; and the association with the personal-social dimension was marginal. Gross and fine motor skills also correlated with subjective stress: higher PNMS resulted in lower scores on these scales. However, there was no relationship between ASQ-3 scores and maternal cognitive appraisal of the flood; and no significant correlations involving the communication scale.

**Table 3:** Correlations between prenatal maternal stress variables and Ages and Stages-3 (ASQ-3) scores

ASQ-3 Scale	Objective Stress	Subjective Stress	Cognitive Appraisal
Communication	−.11	.03	−.04
Gross Motor	−.32**	−.26**	.10
Fine Motor	−.23*	−.20*	.12
Problem Solving	−.19*	−.06	.08
Personal-Social	−.07^§^	−.09	.06

**p < .001; * *p* < .05; ^§^ *p* < .1

### Comparison of care type

**Fig. 2:**
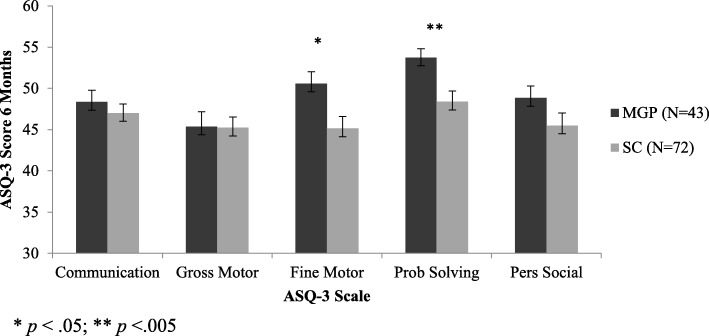
Comparison between the scores on the Ages and Stages Questionnaire (ASQ-3) scales for infants whose mothers received Midwifery Group Practice (MGP) or Standard Care (SC) in pregnancy, when controlling for the effects of flood-related objective hardship

As shown in Fig. 2, ANCOVAS controlling for objective hardship revealed that the MGP group ASQ-3 scores were significantly better than the SC group on the fine motor (*F*(1, 112) = 6.11, *p* = .015) and problem solving (*F*(1, 112) = 7.87, *p* = .006) scales. However, there were no significant differences between MGP and SC on the communication (*F*(1, 112) = 0.52, *p* = 0.48), gross motor (*F*(1, 112) = 0.003, *p* = .96), or personal-social (*F*(1, 112) = 2.43, *p* = .12) scales.

### Regression analyses

The hierarchical regression models showed no significant main effects or interactions between the PNMS measures and model of care when predicting infants’ scores on the communication, gross motor, or personal-social scales (data not shown). However, the final trimmed models for the fine motor and problem solving scales were significant (see Table 4).

**Table 4:** Trimmed hierarchical regression analyses for the Ages and Stages-3 fine motor and problem solving scales

Predictor Variables	*β*	*B*	*Std. Error*	*R*	*R* ^*2*^	*∆R* ^*2*^	*F*	*∆F*
a) Fine Motor								
Step 1				0.231	0.053	0.053	6.376*	6.376*
Objective Stress	−0.231*	−3.442	1.363					
Step 2				0.320	0.102	0.049	6.391**	6.117*
Objective Stress	−0.224*	−3.337	1.334					
Model of Care	0.222*	5.273	2.132					
Step 3				0.353	0.125	0.022	5.278**	2.842^§^
Objective Stress	−0.191*	−2.841	1.355					
Model of Care	0.227*	5.397	2.116					
Depression	−0.153^§^	−0.433	0.257					
b) Problem Solving								
Step 1				0.190	0.036	0.036	4.234*	4.234*
Objective Stress	−0.190*	−2.462	1.197					
Step 2				0.315	0.099	0.063	6.180**	7.868**
Objective Stress	−0.182*	−2.359	1.162					
Model of Care	0.252**	5.212	1.858					
Step 3				0.385	0.149	0.049	6.453***	6.404*
Objective Stress	−0.175*	−2.264	1.136					
Model of Care	0.204*	4.217	1.857					
Birthweight	0.227*	0.005	0.002					

***p < .001; **p < .01; * *p* < .05; ^*§*^ *p* < .1 Model of care: 0 = SC, 1 = MGP

Objective stress explained 5.3% of variance in infants’ fine motor skill: the higher the mother’s flood-related stress, the poorer the infant scored on this scale. In step 2, adding model of care explained a significant additional 4.9% of variance, suggesting that infants in the MGP group attained higher fine motor scores than did infants in the SC group. Finally, post-natal EPDS score accounted for another 2.2% of variance in fine motor skill, with higher EPDS scores marginally associated with poorer fine motor skills. These three variables explained a total of 12.5% of the variance in infant fine motor skills.

Objective stress explained 3.6% of variance in infants’ problem solving: the higher the mothers’ objective stress the poorer the infants scored. In step 2, adding model of care explained a significant additional 6.3% of variance, suggesting that infants in the MGP attained higher problem solving scores than did infants in SC. Finally, infant birth weight accounted for another significant 4.9% of variance, with higher birthweights associated with better problem-solving skills. These three variables explained a total of 14.9% of the variance in problem-solving scores.

### Risk for developmental delay

Chi-squared tests showed that infants in the SC group were significantly more likely than those in MGP to fall at least 1*SD* below the mean on the fine motor (9% vs. 29%) and problem solving scales (2% vs. 15%). However, there were no differences in clinical risk between the two models of care for the communication, gross motor, or personal-social scales (see Table 5).

**Table 5:** Frequency of infants identified as normally developing or requiring monitoring for risk of developmental delay (< 1 *SD* Mean) on the Ages and Stages Questionnaire-3 (ASQ-3) in Midwifery Group Practice (MGP) and Standard Care (SC)

ASQ-3 Scale	MGP *N* (%)	SC *N* (%)	Chi-Sq P value
Communication			0.62
Normal	37 (86.0)	58 (80.6)	
At Risk	6 (14)	14 (19.4)	
Gross Motor			1.00
Normal	37 (86.0)	61 (84.7)	
At Risk	6 (14.0)	11 (15.3)	
Fine Motor			0.02
Normal	39 (90.7)	51 (70.8)	
At Risk	4 (9.3)	21 (29.2)	
Problem Solving			0.03^a^
Normal	42 (97.7)	61 (84.7)	
At Risk	1 (2.3)	11 (15.3)	
Personal-Social			0.86
Normal	36 (83.7)	58 (80.6)	
At Risk	7 (16.3)	14 (19.4)	

^a^Fisher’s Exact Test (2-sided)

## Discussion

In comparing MGP and SC models of maternity care, this study extends the known benefits of continuity of midwifery carer [1–3, 21, 22] to infant neurodevelopment. To our knowledge, this is the first study to show that the MGP model of midwifery care positively influences aspects of infant neurodevelopment in a situation when their mothers had been flood-affected in pregnancy. There were significantly better outcomes for infants whose mothers received MGP care over SC on fine motor and problem-solving scales, even when controlling for the severity of their mothers’ objective hardship from the flood. These effects were also seen at a clinical level with three times as many infants in the SC group met criteria for “monitoring risk” status compared to MGP group. However, there were no significant differences between infants in the MGP and SC groups for the communication, gross motor or personal-social scales.

We tested the ability of MGP to buffer the unborn children from their mothers’ objective and subjective stress. However, because none of the PNMS-by-model of care interactions were significant, we conclude that all infants were negatively influenced by maternal flood-related hardship irrespective of model of care, but that MGP had an independent, positive effect. This research adds to a small number of studies showing that prenatal interventions (e.g., meditation and mindfulness) for stressed or anxious pregnant women can reduce the harmful effects of PNMS on infant development [23, 24], although not in a specifically “buffering” pattern of effects.

The current results also support research from the longitudinal Nurse-Family Partnership program, which provides evidence that regular prenatal home visits by nurses to low-income first time mothers positively influences child development [4]. Childhood assessments also indicated higher intellectual and language outcomes [25–27], and better school academic adjustment [28], for nurse-visited children compared to those from paraprofessionals. The current study extends this work by demonstrating that the infants of economically advantaged women (as in this sample) can also benefit from a model of maternity care which emphasizes continuity of carer.

We hypothesize that the benefits of MGP over SC were primarily due to the relational component of the partnership that developed between women and their midwives across the maternity continuum [1–3, 29]. Women in the MGP model had 24/7 access to a small group of known midwives and hence, had opportunities to build relationships of trust and support not possible in the SC model. In the free text analysis of the M@NGO RCT, MGP women reported being more ‘at ease, comfortable, confident, loved, reassured, relaxed, safe, and supported’ in this model of care compared to women in SC models [29]. The home visits up to 6-weeks postnatally may have also played an important role in supporting women’s transitions to motherhood. Prior QF2011 results demonstrate higher objective and subjective PNMS predicted more severe 6-week postpartum anxiety and depression in the SC mothers, there were no such associations in MGP mothers [22]. These benefits of MGP care over SC for flood-affected pregnant women’s postnatal wellbeing may also have beneficial flow-on effects for enhancing infant neurodevelopment, as seen here.

Note that other variables known to influence infant neurodevelopment, such as maternal demographics and mental health [30, 31], did not vary between the groups. Although, MGP infants had significantly younger mothers, longer gestations, and were heavier than SC infants, these variables were either unrelated to ASQ-3 scores, or the results were unchanged when entered into the regressions. Women in MGP were more likely to be parimiparous than women in SC; but again, this was also unrelated to ASQ-3 scores. As the M@NGO eligibility criteria excluded women with a preference for MGP, many women having a second or subsequent baby often selected MGP, leaving a higher than normal proportion of primiparous women without a preference and therefore eligible to be randomly assigned. As a result, the majority of MGP women were primiparous.

The study was not without limitations. Although the sample size was small, significant between group effects were detectable. The design for the QF2011 study was a prospective cohort study rather than a RCT, so these women (not from the M@NGO RCT) may have self-selected into the MGP or SC model. Thus, the QF2011 women who self-selected MGP may have differed from the women who self-selected SC on some dimension(s). However, we were unable to test for these differences due to uneven distribution between the groups (9 in MGP vs 50 in SC). In QF2011, women were allocated to model of care prior to study enrollment, thus women were unable to be randomized, and self-selection into the model may have introduced a bias that we were unable to detect; and this may have influenced the current results. We encourage replication of the study with more even group numbers. Furthermore, caution must be taken when interpreting the results as the ASQ-3 relied on maternal report of child development, which may be prone to bias or inaccuracy. However, the psychometric properties of the ASQ-3 show that it has high validity with other clinician-administered tools [12]. None-the-less, these findings would be strengthened with an independent observation of child development at older ages by a researcher blind to study hypotheses and model of maternity care showing ongoing positive effects of MGP.

## Conclusions

Optimizing early childhood development is a World Health Organization priority and a focus of major public health campaigns in many developed counties (e.g., Head Start in the USA and Sure Start in the UK). Early interventions are known to be effective in protecting high-risk children from adverse developmental outcomes [32]. This research shows that continuity of midwifery carer is an effective way to reduce the harmful effects of disaster-related stress in pregnancy on aspects of infant neurodevelopment. Whether these early benefits of MGP endure over the long-term will be assessed as we track the development of the QF2011 cohort.

## Abbreviations

ASQ-3: Ages and Stages Questionniare
COSMOSS: Composite Score for Mothers’ Subjective Stress
EPDS: Edinburgh Postnatal Depression Scale
IES-R: Impact of Event Scale – Revised
MGP: Midwifery Group Practice
PCA: Principal Component Analysis
PDEQ: Peritraumatic Dissossiation Experiences Questionnaire
PDI: Peritraumatic Distress Inventory
PNMS: Prenatal maternal stress
QF2011: Queensland Flood Study
QFOSS: Queensland Flood Objective Stress Scale
RCT: Randomized Control Trial
SC: Standard Hospital Care
SEIFA: Socio Economic Indexes For Areas

## References

[1] Tracy SK (2013). Caseload midwifery care versus standard maternity care for women of any risk: M@NGO, a randomised controlled trial. Lancet.

[2] Allen J (2015). Does model of maternity care make a difference to birth outcomes for young women? A retrospective cohort study. Int J Nurs Stud.

[3] Sandall, J., et al., Midwife-led continuity models versus other models of care for childbearing women*.* Cochrane Database Syst Rev, 2016. 4: p. Cd004667. 10.1002/14651858.CD004667.pub5 PMC8663203 27121907

[4] Olds DL (2006). The nurse–family partnership: an evidence-based preventive intervention. Infant Mental Health Journal.

[5] Dancause KN (2011). Disaster-related prenatal maternal stress influences birth outcomes: Project Ice Storm. Early Human Development.

[6] Lilliecreutz C (2016). Effect of maternal stress during pregnancy on the risk for preterm birth. BMC Pregnancy and Childbirth.

[7] Davis EP, Sandman CA (2010). The timing of prenatal exposure to maternal cortisol and psychosocial stress is associated with human infant cognitive development. Child Dev.

[8] Laplante DP (2008). Project ice storm: prenatal maternal stress affects cognitive and linguistic functioning in 5½-year-old children. J Am Acad Child Adolesc Psychiatry.

[9] Simcock G (2016). Age-related changes in the effects of stress in pregnancy on infant motor development by maternal report: the Queensland flood study. Dev Psychobiol.

[10] Davis EP (2004). Prenatal maternal anxiety and depression predict negative behavioral reactivity in infancy. Infancy.

[11] King S (2015). QF2011: a protocol to study the effects of the Queensland flood on pregnant women, their pregnancies, and their children's early development. BMC Pregnancy Childbirth.

[12] Squires J, Twombly E, Bricker D, Potter L (2009). Ages and Stages Questionnaires User's Guide.

[13] Simcock G, et al. Infant neurodevelopment is affected by prenatal maternal stress: The QF2011 flood study. Infancy. 2016:1–22. 10.1111/infa.12166 33158359

[14] Tracy SK, et al. A randomised controlled trial of caseload midwifery care: M@NGO (midwives @ new group practice options). BMC Pregnancy and Childbirth. 2011;11(1):82–2. 10.1186/1471-2393-11-82 PMC3235961 22029746

[15] Yong Ping E (2015). Prenatal maternal stress predicts stress reactivity at 21/2 years: the Iowa flood study. Psychoneuroendocrinology.

[16] Weiss DS, Marmar CR, Wilson JP, Keane TM (1997). The Impact of Event Scale - Revised. Assessing psychological trauma and PTSD: A practitioner's handbook.

[17] Brunet A (2001). The Peritraumatic distress inventory: a proposed measure of PTSD criterion A2. Am J Psychiatr.

[18] Marmar CR, Weiss DS, Metzler TJ, Wilson JP, Keane TM (1997). The Peritraumatic Dissociative Experiences Questionnaire, in Assessing Psychological Trauma and PTSD.

[19] Bricker D (1999). Ages & stages questionnaire: a parent-completed monitoring system.

[20] Cox JL, Holden JM, Sagovsky R (1987). Detection of postnatal depression: development of the 10-item Edinburgh postnatal depression scale. Br J Psychiatry.

[21] McLachlan HL (2012). Effects of continuity of care by a primary midwife (caseload midwifery) on caesarean section rates in women of low obstetric risk: the COSMOS randomised controlled trial. BJOG Int J Obstet Gynaecol.

[22] Kildea S, et al. Continuity of midwifery carer moderates the effects of prenatal maternal stress on postnatal maternal wellbeing: the Queensland flood study. Archives of Womens Ment Health. 2017;2017 10.1007/s00737-017-0781-2 28956168

[23] Chan KP (2014). Prenatal meditation influences infant behaviors. Infant Behavior and Development.

[24] van den Heuvel MI (2015). Maternal mindfulness during pregnancy and infant socio-emotional development and temperament: the mediating role of maternal anxiety. Early Hum Dev.

[25] Olds DL (2002). Home visiting by paraprofessionals and by nurses: a randomized, controlled trial. Pediatrics.

[26] Olds DL (2004). Effects of home visits by paraprofessionals and by nurses: age 4 follow-up results of a randomized trial. Pediatrics.

[27] Olds DL (2004). Effects of nurse home-visiting on maternal life course and child development: age 6 follow-up results of a randomized trial. Pediatrics.

[28] Olds DL (2007). Effects of nurse home visiting on maternal and child functioning: Age-9 follow-up of a randomized trial. Pediatrics.

[29] Allen J (2017). The motivation and capacity to go ‘above and beyond’: qualitative analysis of free-text survey responses in the M@NGO randomised controlled trial of caseload midwifery. Midwifery.

[30] Grace SL, Evindar A, Stewart DE (2003). The effect of postpartum depression on child cognitive development and behavior: a review and critical analysis of the literature. Archives of Womens Mental Health.

[31] Tough SC (2010). Maternal well-being and its association to risk of developmental problems in children at school entry. BMC Pediatr.

[32] Hertzman C, Wiens M (1996). Child develoment and long-term outcomes: a population health perspective and summary o f succesful interventions. Soc Sci Med.

